# Survival Benefit and Safety of Anatomic Resection in Cirrhotic Hepatocellular Carcinoma: Propensity‐Matched Analysis of 1699 Patients

**DOI:** 10.1002/cam4.71537

**Published:** 2026-01-23

**Authors:** Ao Du, Hongwei Xu, Chuang Jiang, Kefei Yuan

**Affiliations:** ^1^ Department of Liver Surgery, State Key Laboratory of Biotherapy and Cancer Center West China Hospital, Sichuan University and Collaborative Innovation Center of Biotherapy Chengdu China; ^2^ Division of Liver Surgery, Department of General Surgery West China Hospital, Sichuan University Chengdu China

**Keywords:** anatomic resection, cirrhosis, hepatocellular carcinoma, non‐anatomic resection, prognosis, propensity score matching

## Abstract

**Background:**

The efficacy of anatomic resection (AR) versus non‐anatomic resection (NAR) for hepatocellular carcinoma (HCC) remains controversial, particularly among patients with differing underlying liver conditions. This study aimed to compare the outcomes of AR and NAR in HCC patients with and without liver cirrhosis.

**Methods:**

We retrospectively analyzed data from HCC patients who underwent hepatectomy at West China Hospital between 2010 and 2022. Patients were stratified based on the presence of liver cirrhosis. Propensity score matching (PSM) was then applied separately within each stratum (cirrhotic and non‐cirrhotic) to compare perioperative outcomes and long‐term prognosis between AR and NAR.

**Results:**

A total of 1699 patients were enrolled, including 866 with cirrhosis and 833 without cirrhosis. Before PSM, AR was associated with significantly superior overall survival (OS) and recurrence‐free survival (RFS) compared to NAR. Following PSM (1:1 matching based on resection type within each cirrhosis stratum), similar survival advantages for AR were observed. In the matched cirrhotic cohort (255 patients per group), the AR group exhibited significantly better OS (median: 36.5 vs. 25.2 months; *p* < 0.001) and RFS (median: 26.9 vs. 17.4 months; *p* < 0.001) compared to the NAR group. Similarly, in the matched non‐cirrhotic cohort (284 patients per group), the AR group showed significantly improved OS (median: 36.3 vs. 23.9 months; *p* < 0.001) and RFS (median: 27.8 vs. 18.5 months; *p* < 0.001) compared to the NAR group. No significant differences were observed in perioperative outcomes, including postoperative complications and hospital length of stay.

**Conclusions:**

Compared to NAR, AR improved long‐term prognosis in HCC patients. This survival benefit was evident even in cirrhotic patients and was not associated with increased perioperative risks.

**Abbreviations**
AFP: α‐fetoprotein
AR: anatomical resection
ASA: American Society of Anesthesiologists
BMI: body mass index
HBV: hepatitis B virus
HCC: hepatocellular carcinoma
HCV: hepatitis C virus
MVI: microvascular invasion
NAFLD: non‐alcoholic fatty liver disease
NAR: non‐anatomic resection
OS: overall survival
PSM: propensity score matching
RFS: recurrence‐free survival

## 1 Introduction

Hepatocellular carcinoma (HCC) is a leading cause of cancer‐related mortality worldwide, and its incidence continues to rise [1, 2, 3]. Treatment modalities for HCC include surgical resection, liver transplantation, local ablation, among others [4]. Partial hepatectomy is a cornerstone treatment for early‐stage HCC, significantly improving patient prognosis [5, 6]. Generally, liver resection encompasses two principal approaches: anatomic resection (AR) and non‐anatomic resection (NAR). AR, first introduced by Makuuchi et al. in 1985 [7], involves the resection of one or more anatomically defined liver segments along with their corresponding portal pedicles. AR is hypothesized to reduce tumor recurrence by eliminating microscopic satellite lesions and portal venous tumor thrombi, thereby improving survival outcomes [8, 9]. In contrast, NAR entails tumor excision with a margin of normal parenchyma, prioritizing maximal preservation of functional liver tissue to theoretically reduce the risk of postoperative liver failure and complications [10]. Despite numerous studies reporting comparative data, the optimal surgical approach (AR vs. NAR) for HCC remains controversial [11, 12, 13]. While most studies suggest that AR improves long‐term survival in HCC patients, others report no significant advantage over NAR [14, 15].

In China, hepatitis B virus (HBV) infection is the predominant etiology of HCC, frequently leading to chronic hepatitis, liver cirrhosis, and ultimately HCC [16]. Cirrhosis, characterized by architectural distortion of the liver due to chronic injury, severely impairs hepatic functional reserve and regenerative capacity. Consequently, hepatic resection in cirrhotic patients poses significant challenges due to an elevated risk of postoperative complications, including liver failure [17]. Although AR has demonstrated superior long‐term survival compared to NAR in some studies, its wider resection extent raises concerns regarding increased perioperative risks, particularly in cirrhotic livers [18, 19, 20]. However, evidence specifically addressing the perioperative safety of AR in cirrhotic HCC patients remains limited. Furthermore, in the era of personalized oncology, understanding how underlying liver disease influences surgical outcomes is crucial. Although AR may confer superior long‐term survival, preserving sufficient future liver remnant (FLR) volume is paramount, especially for cirrhotic patients with compromised hepatic reserve. Therefore, a comprehensive analysis comparing the long‐term prognosis and perioperative outcomes of AR versus NAR across different liver disease backgrounds is essential to guide optimal surgical strategy selection.

This study aimed to retrospectively compare the long‐term survival and perioperative outcomes of AR versus NAR in HCC patients with and without liver cirrhosis. The findings may provide valuable insights for balancing survival benefits against perioperative risks, thereby informing personalized surgical decision‐making in HCC management.

## 2 Patients and Methods

### 2.1 Patients

We retrospectively analyzed data from consecutive HCC patients who underwent curative‐intent hepatic resection at West China Hospital between January 2010 and December 2022. Based on the inclusion criteria—(1) histologically confirmed HCC; (2) Child‐Pugh class A or B; and (3) R0 resection—a total of 1699 patients were enrolled. Exclusion criteria comprised: (1) incomplete clinicopathological data; (2) preoperative extrahepatic metastasis; (3) concurrent other malignancies; and (4) emergency surgery. All patients provided written informed consent for the scientific use of their anonymized clinical data. This study was conducted in accordance with the Declaration of Helsinki and approved by the Ethics Committee of West China Hospital, Sichuan University.

### 2.2 Outcomes and Data Collection

Data were retrospectively extracted from the electronic medical record system, including: demographic information such as age and gender from patient profiles, comorbidities (e.g., hypertension, diabetes, cardiovascular diseases) from admission records, HBV/HCV infection status from medical history, surgical‐related data such as intraoperative blood loss and operation time from surgical records, perioperative complications from clinical progress notes, liver cirrhosis, fatty liver, tumor size, tumor number, microvascular invasion (MVI) status, macrovascular invasion and resection margins from imaging and pathology reports, and alpha‐fetoprotein (AFP) levels from laboratory tests. Survival data were obtained from the institutional follow‐up database. Postoperative complications were recorded according to the Clavien‐Dindo classification [21].

AR was defined as the complete resection of anatomically defined territories (subsegments, segments, or sectionectomies) based on the portal venous inflow, including the tumor‐bearing portal pedicles. Resections not meeting this anatomical definition were classified as NAR [15].

### 2.3 Follow‐Up

Follow‐up was primarily conducted through outpatient clinics. Active follow‐up occurred every 3 months during the first 2 years postoperatively and every 6 months thereafter. Patients who missed follow‐up appointments were contacted via telephone by research assistants. Overall survival (OS) was defined as the interval from surgery to death from any cause or the last follow‐up. Recurrence‐free survival (RFS) was defined as the interval from surgery to the first recurrence (local or distant), death from any cause, or the last follow‐up.

### 2.4 Statistical Analysis

Continuous variables are expressed as median with interquartile range (IQR), while categorical variables are presented as frequencies and percentages. Between‐group comparisons for continuous variables were performed using the Student's *t*‐test or Mann–Whitney *U* test, as appropriate. Categorical variables were compared using the *χ* ^2^ test or Fisher's exact test, as appropriate. Propensity score matching (PSM) was employed to balance baseline characteristics between the AR and NAR groups within each cirrhosis stratum (cirrhotic and non‐cirrhotic). The propensity score was estimated using all the pre‐operative patient and tumor characteristics listed in Table 1. Matching was then performed using a 1:1 nearest‐neighbor algorithm with a caliper width of 0.2 standard deviations of the logit of the propensity score. Match quality was assessed using standardized mean differences (SMD), with a post‐matching SMD < 0.1 for all covariates considered indicative of adequate balance. To evaluate the potential impact of unmeasured confounding, Rosenbaum bounds sensitivity analysis was performed. The robustness of results was assessed by calculating adjusted t‐values at different gamma (*Γ*) levels, with an adjusted *t*‐value ≥ 1.96 (corresponding to *Γ* > 2.0) considered indicative of robust findings. Survival outcomes were estimated using the Kaplan–Meier method and compared between groups with the log‐rank test. All statistical analyses were performed using R software (version 4.4.1; R Foundation for Statistical Computing, Vienna, Austria). A two‐sided *p*‐value < 0.05 was considered statistically significant.

**TABLE 1:** Baseline Clinicopathological Characteristics Among Different Groups Before PSM.

Variables	Cirrhotic patients (*n* = 866)				Non‐cirrhotic patients (*n* = 833)			
	NAR (*n*, %)	AR (*n*, %)	*p*	SMD	NAR (*n*, %)	AR (*n*, %)	*p*	SMD
Gender, *n* (%)			1.000	0.108			0.359	0.104
Male	483 (84.15)	246 (84.25)			439 (86.59)	274 (84.05)		
Female	91 (15.85)	46 (15.75)			68 (13.41)	52 (15.95)		
Age (years), *n* (%)			0.285	0.202			0.069	0.106
≤ 60	434 (75.61)	231 (79.11)			345 (68.05)	201 (61.66)		
> 60	140 (24.39)	61 (20.89)			162 (31.95)	125 (38.34)		
BMI (kg/m^2^), (median (IQR))	23.04 (20.84, 25.63)	22.84 (20.44, 24.80)	0.084	−0.140	23.01 (20.92, 25.29)	22.84 (20.81, 24.96)	0.388	−0.130
Diabetes			0.837	0.001			0.815	0.027
No	531 (92.51)	272 (93.15)			464 (91.52)	296 (90.80)		
Yes	43 (7.49)	20 (6.85)			43 (8.48)	30 (9.20)		
Hypertension, *n* (%)			1.000	0.058			0.581	0.062
No	494 (86.06)	251 (85.96)			424 (83.63)	267 (81.90)		
Yes	80 (13.94)	41 (14.04)			83 (16.37)	59 (18.10)		
Cardiovascular disease, *n* (%)			1.000	0.084			1.000	0.060
No	569 (99.13)	289 (98.97)			495 (97.63)	318 (97.55)		
Yes	5 (0.87)	3 (1.03)			12 (2.37)	8 (2.45)		
HBV infection, *n* (%)			0.954	0.052			0.120	0.129
No	47 (8.19)	25 (8.56)			119 (23.47)	93 (28.53)		
Yes	527 (91.81)	267 (91.44)			388 (76.53)	233 (71.47)		
HCV infection, *n* (%)			0.438	0.182			1.000	0.094
No	565 (98.43)	290 (99.32)			498 (98.22)	321 (98.47)		
Yes	9 (1.57)	2 (0.68)			9 (1.78)	5 (1.53)		
Alcohol, *n* (%)			0.112	0.089			0.189	0.006
No	334 (58.19)	187 (64.04)			303 (59.76)	179 (54.91)		
Yes	240 (41.81)	105 (35.96)			204 (40.24)	147 (45.09)		
NAFLD, *n* (%)			0.068	0.284			0.055	0.235
No	528 (91.99)	279 (95.55)			463 (91.32)	310 (95.09)		
Yes	46 (8.01)	13 (4.45)			44 (8.68)	16 (4.91)		
AFP, *n* (%)			0.512	0.095			0.392	0.048
≤ 400 ng/mL	476 (82.93)	248 (84.93)			423 (83.43)	280 (85.89)		
> 400 ng/mL	98 (17.07)	44 (15.07)			84 (16.57)	46 (14.11)		
ALT (U/L), (median (IQR))	37.00 (26.000, 53.00)	34.50 (24.000, 49.25)	0.090	−0.382	36.00 (24.00, 56.00)	34.50 (22.00, 52.00)	0.150	−0.025
AST (U/L), (median (IQR))	37.00 (28.00, 52.00)	37.00 (29.00, 52.00)	0.637	−0.229	36.00 (27.00, 56.00)	36.00 (27.00, 55.00)	0.809	0.051
Child‐Pugh stage, *n* (%)			0.911	0.117			1.000	0.046
A	570 (99.30)	289 (98.97)			503 (99.21)	323 (99.08)		
B	4 (0.70)	3 (1.03)			4 (0.79)	3 (0.92)		
Hepatectomy range, *n* (%)			0.321	0.059			0.115	0.056
Minor	466 (81.18)	228 (78.08)			333 (65.68)	232 (71.17)		
Major	108 (18.82)	64 (21.92)			174 (34.32)	94 (28.83)		
Laparoscope, *n* (%)			0.810	0.007			0.078	0.139
No	477 (83.10)	240 (82.19)			407 (80.28)	244 (74.85)		
Yes	97 (16.90)	52 (17.81)			100 (19.72)	82 (25.15)		
ASA classification, *n* (%)			0.069				0.196	
1	401 (69.86)	212 (72.60)		0.106	325 (64.10)	190 (58.28)		−0.077
2	148 (25.78)	76 (26.03)		−0.019	162 (31.95)	124 (38.04)		0.064
3	25 (4.36)	4 (1.37)		−0.460	20 (3.94)	12 (3.68)		0.036
Tumor size, *n* (%)			< 0.001	0.235			0.045	0.050
≤ 5 cm	368 (64.11)	146 (50.00)			233 (45.96)	126 (38.65)		
> 5 cm	206 (35.89)	146 (50.00)			274 (54.04)	200 (61.35)		
Tumor number, *n* (%)			0.030	0.132			< 0.001	0.371
Single	453 (78.92)	249 (85.27)			415 (81.85)	303 (92.94)		
Multiple	121 (21.08)	43 (14.73)			92 (18.15)	23 (7.06)		
MVI, *n* (%)			0.010	0.017			0.398	0.115
No	416 (72.47)	186 (63.70)			362 (71.40)	223 (68.40)		
Yes	158 (27.53)	106 (36.30)			145 (28.60)	103 (31.60)		
Macrovascular invasion, *n* (%)			0.634	0.015			0.942	0.009
No	526 (91.64)	264 (90.41)			467 (92.11)	299 (91.72)		
Yes	48 (8.36)	28 (9.59)			40 (7.89)	27 (8.28)		
Satellite, *n* (%)			0.712	0.010			0.856	0.020
No	521 (90.77)	262 (89.73)			465 (91.72)	297 (91.10)		
Yes	53 (9.23)	30 (10.27)			42 (8.28)	29 (8.90)		

Abbreviations: AFP, α‐fetoprotein; ALT, alanine aminotransferase; AR, anatomical resection; ASA, American Society of Anesthesiologists; AST, aspartate aminotransferase; BMI, body mass index; HBV, hepatitis B virus; HCV, hepatitis C virus; MVI, microvascular invasion; NAFLD, non‐alcoholic fatty liver disease; NAR, nonanatomical resection; SMD, standard mean difference.

## 3 Results

Among the identified 1788 cHCC‐CCA patients, 1699 met our inclusion criteria and were enrolled in this study (Figure S1).

### 3.1 Baseline Characteristics of the Entire Study Cohort

The study cohort comprised 1699 HCC patients. The majority were male (84.9%), with a median age of 53.0 years (IQR: 45.0–62.0 years). AR was performed in 618 patients (36.4%), while NAR was performed in 1081 patients (63.6%). Cirrhosis was present in 866 patients (51.0%); 833 patients (49.0%) had no cirrhosis. Hepatitis B virus (HBV) infection was present in the majority of patients (83.3%), reflecting the predominant etiology of HCC in China. Detailed baseline characteristics of the entire cohort are presented in Table S1.

### 3.2 Long‐Term Prognosis of AR Versus NAR in Different Liver Conditions

**FIGURE 1:**
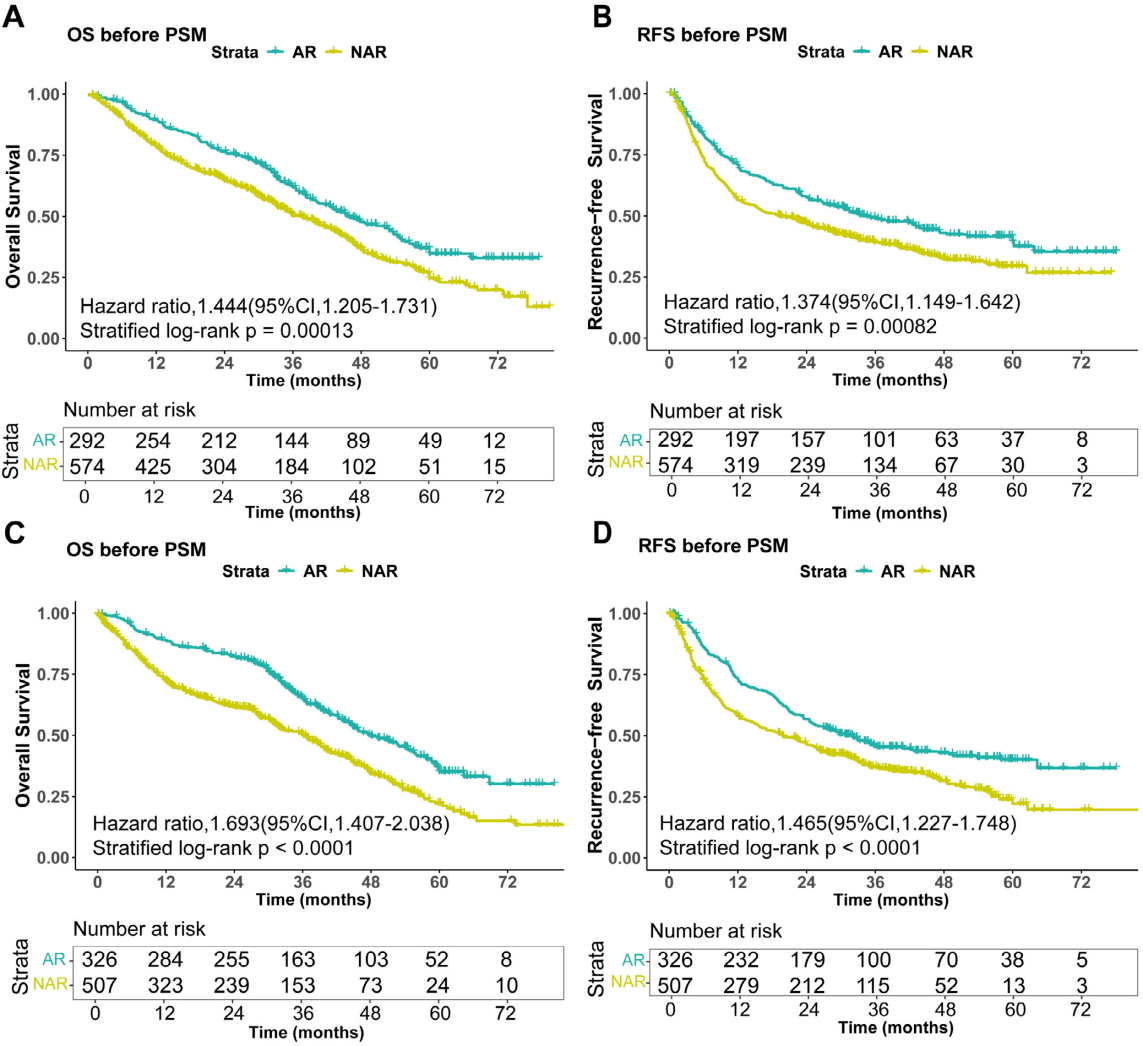
Overall survival and recurrence‐free survival curves for AR and NAR in cirrhotic and non‐cirrhotic cohorts before PSM. (A) Overall survival curves for AR and NAR in the cirrhotic cohort; (B) Recurrence‐free survival curves for AR and NAR in the cirrhotic cohort; (C) Overall survival curves for AR and NAR in the non‐cirrhotic cohort; (D) Recurrence‐free survival curves for AR and NAR in the non‐cirrhotic cohort. AR, anatomical resection; NAR, non‐anatomic resection.

Patients were stratified into two cohorts based on cirrhosis status: cirrhotic (*n* = 866) and non‐cirrhotic (*n* = 833). Outcomes of AR and NAR were compared within each cohort. In the cirrhotic cohort, the median OS was significantly longer in the AR group than in the NAR group (35.55 vs. 26.65 months; *p* < 0.001). Similarly, median RFS was longer with AR (26.85 vs. 17.20 months; *p* < 0.001). In the non‐cirrhotic cohort, AR was also associated with significantly improved median OS (35.90 vs. 22.00 months; *p* < 0.001) and RFS (27.65 vs. 16.20 months; *p* < 0.001) compared to NAR. Thus, prior to PSM, AR demonstrated superior survival outcomes compared to NAR in both cirrhotic and non‐cirrhotic patients (Figure 1). Baseline characteristics of the AR and NAR subgroups within each cirrhosis cohort before PSM are detailed in Table 1. In the cirrhotic cohort, 292 patients underwent AR and 574 underwent NAR. In the non‐cirrhotic cohort, 326 patients underwent AR and 507 underwent NAR. Comparison of baseline characteristics revealed imbalances between the AR and NAR groups, as indicated by observed differences in tumor size and number (*p* < 0.05), alongside standardized mean differences (SMD) greater than 0.1 for several other variables. These discernible differences motivated the use of propensity score matching to mitigate potential confounding.

**FIGURE 2:**
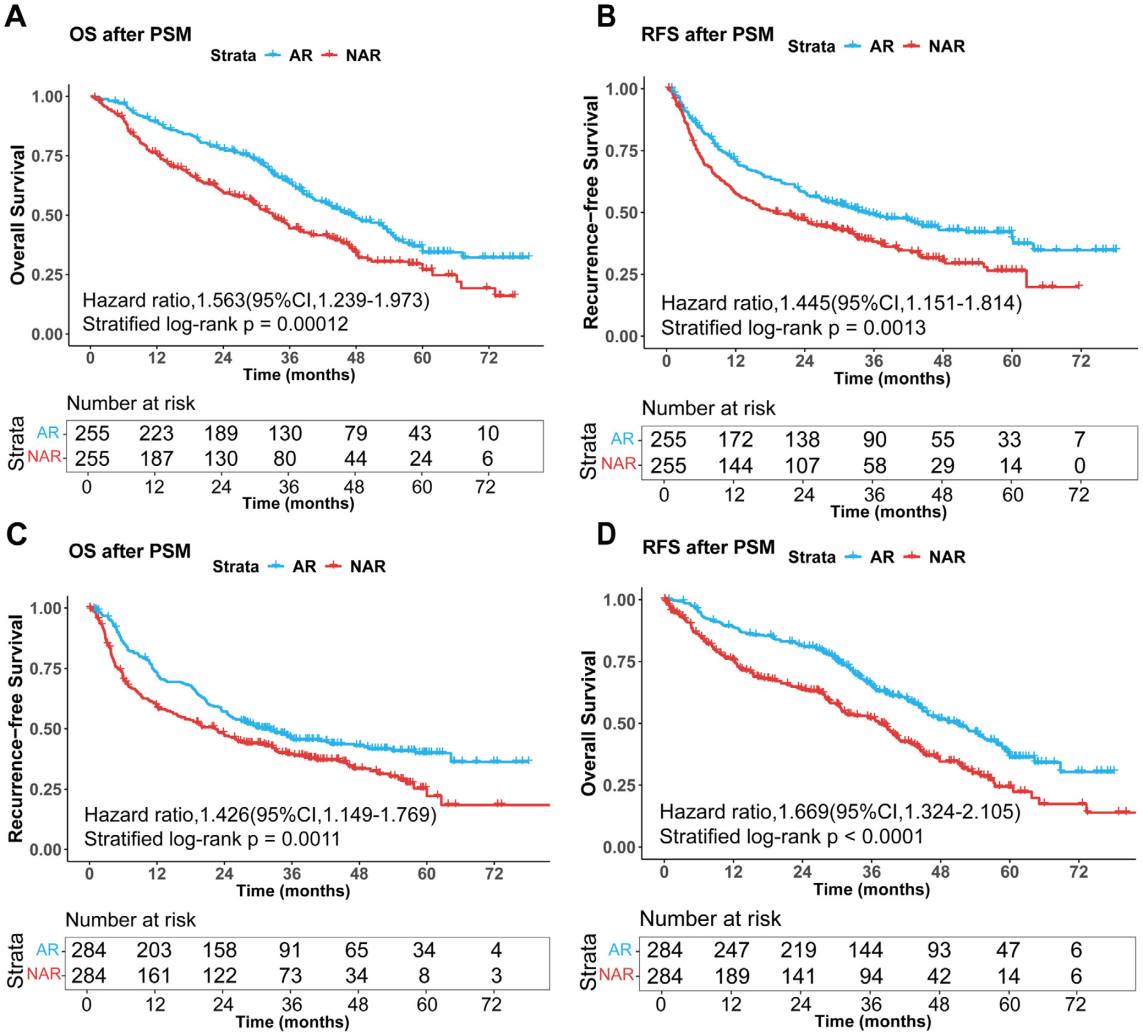
Overall survival and recurrence‐free survival curves for AR and NAR in cirrhotic and non‐cirrhotic cohorts after PSM. (A) Overall survival curves for AR and NAR in the cirrhotic cohort; (B) Recurrence‐free survival curves for AR and NAR in the cirrhotic cohort; (C) Overall survival curves for AR and NAR in the non‐cirrhotic cohort; (D) Recurrence‐free survival curves for AR and NAR in the non‐cirrhotic cohort. AR, anatomical resection; NAR, non‐anatomic resection.

Subsequently, PSM (1:1 ratio) was performed separately within the cirrhotic and non‐cirrhotic cohorts based on resection type. After PSM, the matched cirrhotic cohort comprised 510 patients (255 per group), and the matched non‐cirrhotic cohort comprised 568 patients (284 per group). As detailed in Table 2, baseline characteristics were well‐balanced between AR and NAR groups after matching in both cohorts, including tumor size and number. In the matched cirrhotic cohort, the AR group maintained significantly longer median OS (36.50 vs. 25.20 months; *p* < 0.001) and RFS (26.90 vs. 17.40 months; *p* < 0.001) compared to the NAR group. Similarly, in the matched non‐cirrhotic cohort, AR was associated with superior median OS (36.30 vs. 23.85 months; *p* < 0.001) and RFS (27.80 vs. 18.45 months; *p* < 0.001) compared to NAR. Kaplan–Meier survival curves confirmed significantly better survival outcomes with AR compared to NAR in both matched cohorts, irrespective of cirrhosis status (Figure 2). To evaluate the potential impact of unmeasured confounding factors, we conducted a sensitivity analysis on the propensity score‐matched results. The findings demonstrated that the survival differences between the two groups remained statistically significant even when unmeasured confounders increased the likelihood of treatment selection by more than two‐fold (*Γ* > 2.0). Consistent robustness was observed in both cirrhotic and non‐cirrhotic subgroups (with key *Γ* values > 2.0), indicating that the study conclusions are unlikely to be substantially affected by potential confounding factors (Tables S2 and S3).

**TABLE 2:** Baseline clinicopathological characteristics among different groups after PSM.

Variables	Cirrhotic patients (*n* = 510)				Non‐cirrhotic patients (*n* = 568)			
	NAR (*n*, %)	AR (*n*, %)	*p*	SMD	NAR (*n*, %)	AR (*n*, %)	*p*	SMD
Gender, *n* (%)			1.000	0.030			0.733	0.087
Male	211 (82.75)	212 (83.14)			240 (84.51)	236 (83.10)		
Female	44 (17.25)	43 (16.86)			44 (15.49)	48 (16.90)		
Age (years), *n* (%)			0.174	0.093			1.000	0.028
≤ 60	188 (73.73)	202 (79.22)			182 (64.08)	183 (64.44)		
> 60	67 (26.27)	53 (20.78)			102 (35.92)	101 (35.56)		
BMI (kg/m^2^), (median (IQR))	22.86 (20.95, 25.13)	22.89 (20.55, 24.95)	0.468	−0.005	22.71 (20.83, 24.83)	22.80 (20.82, 25.00)	0.799	−0.010
Diabetes			0.726	0.041			0.645	0.000
No	239 (93.73)	236 (92.55)			259 (91.20)	263 (92.61)		
Yes	16 (6.27)	19 (7.45)			25 (8.80)	21 (7.39)		
Hypertension, *n* (%)			0.403	0.091			1.000	0.076
No	209 (81.96)	217 (85.10)			236 (83.10)	236 (83.10)		
Yes	46 (18.04)	38 (14.90)			48 (16.90)	48 (16.90)		
Cardiovascular disease, *n* (%)			1.000	0.089			0.543	0.086
No	252 (98.82)	252 (98.82)			280 (98.59)	277 (97.54)		
Yes	3 (1.18)	3 (1.18)			4 (1.41)	7 (2.46)		
HBV infection, *n* (%)			0.339	0.035			0.847	0.019
No	25 (9.80)	18 (7.06)			74 (26.06)	71 (25.00)		
Yes	230 (90.20)	237 (92.94)			210 (73.94)	213 (75.00)		
HCV infection, *n* (%)			1.000	0.000			1.000	0.005
No	253 (99.22)	253 (99.22)			280 (98.59)	281 (98.94)		
Yes	2 (0.78)	2 (0.78)			4 (1.41)	3 (1.06)		
Alcohol, *n* (%)			1.000	0.027			0.734	0.062
No	161 (63.14)	160 (62.75)			163 (57.39)	168 (59.15)		
Yes	94 (36.86)	95 (37.25)			121 (42.61)	116 (40.85)		
NAFLD, *n* (%)			1.000	0.013			1.000	0.062
No	242 (94.90)	243 (95.29)			270 (95.07)	269 (94.72)		
Yes	13 (5.10)	12 (4.71)			14 (4.93)	15 (5.28)		
AFP, *n* (%)			1.000	0.092			0.558	0.055
≤ 400 ng/mL	217 (85.10)	216 (84.71)			238 (83.80)	244 (85.92)		
> 400 ng/mL	38 (14.90)	39 (15.29)			46 (16.20)	40 (14.08)		
ALT (U/L), (median (IQR))	37.00 (26.00, 54.00)	35.00 (24.00, 50.00)	0.260	−0.016	35.00 (22.00, 55.00)	35.00 (22.00, 52.25)	0.6532	−0.041
AST (U/L), (median (IQR))	39.00 (28.00, 57.00)	37.00 (29.00, 52.00)	0.557	0.007	36.00 (27.00, 55.00)	35.50 (27.00, 56.50)	0.889	0.012
Child‐Pugh stage, *n* (%)			1.000	0.000			1.000	0.055
A	253 (99.22)	254 (99.61)			280 (98.59)	281 (98.94)		
B	2 (0.78)	1 (0.39)			4 (1.41)	3 (1.06)		
Hepatectomy range, *n* (%)			0.825	0.025			0.854	0.000
Minor	205 (80.39)	202 (79.22)			198 (69.72)	201 (70.77)		
Major	50 (19.61)	53 (20.78)			86 (30.28)	83 (29.23)		
Laparoscope, *n* (%)			0.909	0.028			0.920	0.016
No	207 (81.18)	209 (81.96)			220 (77.46)	222 (78.17)		
Yes	48 (18.82)	46 (18.04)			64 (22.54)	62 (21.83)		
ASA classification, *n* (%)			0.309				0.996	
1	178 (69.80)	180 (70.59)		−0.188	174 (61.27)	173 (60.92)		−0.056
2	69 (27.06)	72 (28.24)		0.191	99 (34.86)	100 (35.21)		0.099
3	8 (3.14)	3 (1.18)		0.000	11 (3.87)	11 (3.87)		−0.109
Tumor size, *n* (%)			0.722	0.000			0.732	0.028
≤ 5 cm	142 (55.69)	137 (53.73)			117 (41.20)	112 (39.44)		
> 5 cm	113 (44.31)	118 (46.27)			167 (58.80)	172 (60.56)		
Tumor number, *n* (%)			0.709	0.030			1.000	0.026
Single	219 (85.88)	215 (84.31)			263 (92.61)	263 (92.61)		
Multiple	36 (14.12)	40 (15.69)			21 (7.39)	21 (7.39)		
MVI, *n* (%)			1.000	0.068			0.410	0.060
No	172 (67.45)	172 (67.45)			204 (71.83)	194 (68.31)		
Yes	83 (32.55)	83 (32.55)			80 (28.17)	90 (31.69)		
Macrovascular invasion, *n* (%)			0.322	0.000			1.000	0.097
No	223 (87.45)	231 (90.59)			259 (91.20)	260 (91.55)		
Yes	32 (12.55)	24 (9.41)			25 (8.80)	24 (8.45)		
Satellite, *n* (%)			1.000	0.035			0.451	0.082
No	232 (90.98)	231 (90.59)			263 (92.61)	257 (90.49)		
Yes	23 (9.02)	24 (9.41)			21 (7.39)	27 (9.51)		

Abbreviations: AFP, α‐fetoprotein; ALT, alanine aminotransferase; AR, anatomical resection; ASA, American Society of Anesthesiologists; AST, aspartate aminotransferase; BMI, body mass index; HBV, hepatitis B virus; HCV, hepatitis C virus; MVI, microvascular invasion; NAFLD, non‐alcoholic fatty liver disease; NAR, nonanatomical resection; SMD, standard mean difference.

### 3.3 Perioperative Outcomes of AR Versus NAR in Different Liver Conditions

To assess whether AR increases perioperative risks, particularly in cirrhosis, we compared perioperative outcomes between AR and NAR within the matched cirrhotic and non‐cirrhotic cohorts. In the matched cirrhotic cohort, no significant differences were observed between AR and NAR groups in terms of intraoperative blood loss (*p* = 0.734), operation time (*p* = 0.608), 90‐day mortality (*p* = 1.000), length of hospital stay (LOS) (*p* = 0.739), ICU admission rate (*p* = 0.479), overall postoperative complication rate (*p* = 0.279), major complication rate (*p* = 1.000), or specific complications such as liver failure, bile leakage, and postoperative hemorrhage; furthermore, no significant differences were found in recurrence site or causes of death between the two groups (Table 3). Similar findings were observed in the matched non‐cirrhotic cohort (Table 3). These results indicate that AR, even in cirrhotic patients, was not associated with an increased risk of postoperative complications compared to NAR.

**TABLE 3:** Surgery and morbidity stratified for patient among different groups after PSM.

Variables	Cirrhotic patients (*n* = 510)			Non‐cirrhotic patients (*n* = 568)		
	NAR (*n*, %)	AR (*n*, %)	*p*	NAR (*n*, %)	AR (*n*, %)	*p*
Intraoperative bleeding (mL), (median (IQR))	200.00 (100.00, 400.00)	200.00 (100.00, 400.00)	0.734	250.00 (150.00, 400.00)	300.00 (162.00, 500.00)	0.111
Operation time (min), (median (IQR))	205.00 (165.00, 260.00)	210.00 (170.00, 265.00)	0.608	205.00 (170.00, 250.00)	220.00 (177.25, 270.00)	0.087
Death within 90 days						
No	247 (96.86)	247 (96.86)	1.000	282 (99.30)	280 (98.59)	0.682
Yes	8 (3.14)	8 (3.14)		2 (0.70)	4 (1.41)	
LOS (days), (median (IQR))	10.00 (8.00, 13.00)	10.00 (8.00, 13.00)	0.739	10.00 (8.00, 13.00)	10.50 (8.00, 13.00)	0.315
ICU, *n* (%)			0.479			0.616
No	253 (99.22)	255 (100.00)		283 (99.65)	281 (98.94)	
Yes	2 (0.78)	0 (0.00)		1 (0.35)	3 (1.06)	
Complication, *n* (%)			0.279			0.625
0	125 (49.02)	122 (47.84)		123 (43.31)	139 (48.94)	
1	101 (39.61)	99 (38.82)		132 (46.48)	117 (41.20)	
2	15 (5.88)	20 (7.84)		19 (6.69)	17 (5.99)	
3	8 (3.14)	13 (5.10)		6 (2.11)	6 (2.11)	
4	4 (1.57)	0 (0.00)		3 (1.06)	5 (1.76)	
5	2 (0.78)	1 (0.39)		1 (0.35)	0 (0.00)	
Major complication, *n* (%)			1.000			0.846
No	241 (94.51)	241 (94.51)		274 (96.48)	273 (96.13)	
Yes	14 (5.49)	14 (5.49)		10 (3.52)	11 (3.87)	
Specific common complications						
Liver failure, *n* (%)	3 (1.18)	3 (1.18)	1.000	3 (1.06)	4 (1.41)	1.000
Pleural effusion, *n* (%)	7 (2.75)	8 (3.14)	1.000	6 (2.11)	4 (1.41)	0.750
Bile leakage, *n* (%)	13 (5.10)	17 (6.67)	0.572	17 (5.99)	25 (8.80)	0.262
Ascites, *n* (%)	1 (0.39)	7 (2.75)	0.075	0 (0.00)	2 (0.70)	0.479
Bleeding, *n* (%)	17 (6.67)	17 (6.67)	1.000	18 (6.34)	15 (5.28)	0.710
Surgical site infection, *n* (%)	3 (1.18)	6 (2.35)	0.501	12 (4.23)	8 (2.82)	0.495
Pulmonary infection, *n* (%)	1 (0.39)	2 (0.78)	1.000	3 (1.06)	1 (0.35)	0.616
Arrhythmia, *n* (%)	17 (6.67)	15 (5.88)	0.855	16 (5.63)	15 (5.28)	1.000
Circulatory disorder, *n* (%)	7 (2.75)	2 (0.78)	0.179	2 (0.70)	3 (1.06)	1.000
Vomiting, *n* (%)	8 (3.14)	17 (6.67)	0.101	18 (6.34)	15 (5.28)	0.720
Systemic infection, *n* (%)	3 (1.18)	3 (1.18)	1.000	3 (1.06)	2 (0.70)	1.000
Recurrence site			0.502			0.386
Intrahepatic recurrence	22 (13.50)	15 (10.95)		44 (24.72)	26 (16.56)	
Pulmonary metastasis	10 (6.13)	6 (4.38)		14 (7.87)	14 (8.92)	
Osseous metastasis	7 (4.29)	5 (3.65)		5 (2.81)	3 (1.91)	
Lymphatic metastasis	6 (3.68)	3 (2.19)		6 (3.37)	3 (1.91)	
Multiple metastases	9 (5.52)	5 (3.65)		15 (8.43)	13 (8.28)	
Metastasis to other organs	6 (3.68)	12 (8.76)		10 (5.62)	6 (3.82)	
NA	103 (63.19)	91 (66.42)		84 (47.19)	92 (58.60)	
The cause of death			0.280			0.593
Liver failure	6 (3.87)	8 (5.88)		1 (0.64)	3 (2.10)	
Recurrence or distant metastasis	95 (61.29)	68 (50.00)		77 (49.36)	65 (45.45)	
Systemic infection	0 (0.00)	1 (0.74)		2 (1.28)	1 (0.70)	
Upper gastrointestinal bleeding	3 (1.94)	2 (1.47)		1 (0.64)	0 (0.00)	
NA	51 (32.90)	57 (41.91)		75 (48.08)	74 (51.75)	

Abbreviations: AR, anatomical resection; LOS, length of stay; NA, not available; NAR, nonanatomical resection.

### 3.4 Recurrence Time of AR Versus NAR in Different Liver Conditions

We further compared early recurrence rates between AR and NAR groups within the matched cirrhotic and non‐cirrhotic cohorts. Results demonstrated a lower probability of early recurrence (within 12 months) in the AR group compared to the NAR group (Table 4). Specifically, in the matched cirrhotic cohort, the 12‐month recurrence rate was significantly lower in the AR group (50.36%) than in the NAR group (65.03%). Similarly, in the matched non‐cirrhotic cohort, the 12‐month recurrence rate was lower with AR (47.77% vs. 63.48%). These findings suggest that AR may reduce the risk of early recurrence compared to NAR.

**TABLE 4:** Time of recurrence in patients among different groups after PSM.

Variables	Cirrhotic patients (*n* = 300)			Non‐cirrhotic patients (*n* = 335)		
	NAR (*n* = 163)	AR (*n* = 137)	*p*	NAR (*n* = 178)	AR (*n* = 157)	*p*
Time to recurrence			0.033			0.013
Recurrence 12 months, *n* (%)	106 (65.03)	69 (50.36)		113 (63.48)	75 (47.77)	
Recurrence in 12–24 months, *n* (%)	26 (15.95)	34 (24.82)		32 (17.98)	44 (28.02)	
Recurrence over 24 months, *n* (%)	31 (19.02)	34 (24.82)		33 (18.54)	38 (24.20)	

Abbreviations: AR, anatomical resection; NAR, nonanatomical resection.

## 4 Discussion

HCC is the predominant form of primary liver cancer and ranks as the third leading cause of cancer‐related mortality worldwide [22, 23]. Major risk factors encompass chronic infection with hepatitis viruses (HBV, HCV), heavy alcohol consumption, non‐alcoholic fatty liver disease (NAFLD), and liver cirrhosis [24, 25]. In China, HBV infection is responsible for the vast majority of HCC cases, and most patients present with underlying cirrhosis [25]. Cirrhosis arises from chronic hepatic injury, leading to variable degrees of architectural distortion and functional impairment. Furthermore, cirrhotic patients frequently develop portal hypertension and its sequelae (e.g., splenomegaly, varices), significantly elevating the risk of complications including liver failure, infection, and hemorrhage [26, 27]. Nevertheless, advances in surgical and perioperative management have established hepatic resection as a feasible option for selected cirrhotic patients [28, 29]. However, greater caution is warranted compared to non‐cirrhotic patients due to the reduced functional reserve and regenerative capacity of the cirrhotic liver, limiting the tolerable resection volume. Consequently, concerns persist regarding the safety of major hepatic resection in cirrhotic patients [30, 31].

The primary surgical approaches for liver tumors are anatomic resection (AR) and non‐anatomic resection (NAR). AR aims for a more oncologically radical resection by removing the tumor along with its anatomical territory (e.g., segment, section), potentially providing wider margins and reducing the risk of recurrence from occult satellite lesions or venous spread [32]. Potential disadvantages of AR include a larger parenchymal resection volume, greater technical complexity, and potentially higher intraoperative blood loss [33]. These factors may pose particular challenges in cirrhotic patients, potentially increasing the risk of postoperative complications due to impaired functional reserve. In contrast, NAR aims to achieve R0 resection where technically and anatomically feasible, without regard for segmental or portal territorial anatomy. Typical approaches include wedge resection for peripheral tumors or limited/elliptical resection for superficially located lesions. In our institution, NAR is routinely performed with intraoperative ultrasonography for precise tumor localization and guidance of parenchymal transection. Although NAR generally preserves more functional liver parenchyma, it may be associated with higher risks of local recurrence and potentially inferior long‐term outcomes, likely attributable to narrower resection margins and incomplete removal of occult micrometastases within the anatomical territory [34].

This dichotomy raises the critical question of whether cirrhotic patients should undergo AR or NAR. However, studies specifically comparing AR and NAR in cirrhotic HCC patients are limited. Our study aimed to address this gap in evidence. Utilizing PSM to mitigate confounding, we analyzed a large cohort from a tertiary referral center in China to compare AR and NAR with respect to long‐term survival (OS, RFS), recurrence patterns, and perioperative outcomes. Our results demonstrate that AR was associated with significantly improved OS compared to NAR, irrespective of underlying cirrhosis status. This finding aligns with numerous studies demonstrating superior long‐term survival with AR compared to NAR in overall HCC cohorts, although many were not focused specifically on cirrhotic patients. For instance, a study by Masayuki et al. of 268 HCC patients utilized PSM and identified AR as an independent favorable prognostic factor (HR = 0.456, *p* = 0.039), with significantly longer median OS in the AR group (76.2 ± 6.3 months) versus the NAR group (58.9 ± 6.3 months; *p* < 0.01) [35]. Similarly, a meta‐analysis of 12 studies reported superior 1‐, 3‐, and 5‐year OS rates for AR over NAR, with a significant overall survival benefit (HR = 0.62, 95% CI: 0.47–0.82; *p* = 0.001) [36]. However, other studies have reported no significant survival difference between AR and NAR. For example, a multicenter study employing PSM found no significant difference in OS between AR and NAR groups based on Kaplan–Meier analysis [37]. Similarly, Kwon et al., in a study of 986 patients, reported that AR conferred a survival benefit only for tumors ≤ 5 cm in diameter (*p* < 0.001), with no advantage observed for larger tumors [38]. Notably, this contrasts with findings from our prior meta‐analysis in intrahepatic cholangiocarcinoma (ICC), where AR demonstrated a greater survival advantage specifically for tumors > 5 cm (HR = 0.73, 95% CI: 0.51–0.87; *p* = 0.003) [39]. These findings suggest that the benefits of AR may be context‐dependent, underscoring the need for further research to refine patient stratification and more accurately identify those most likely to benefit from AR.

The high recurrence rate remains a major contributor to the poor prognosis of HCC [40]. Theoretically, by resecting the anatomical territory, AR enables wider surgical margins and may reduce recurrence risk by eliminating microscopic satellite lesions within the portal venous drainage area. Consequently, the impact of AR on HCC recurrence has been a focus of investigation. In our study, AR was associated with significantly prolonged median RFS compared to NAR (26.00 vs. 16.10 months; *p* < 0.001) and a significantly lower 12‐month recurrence rate (52.71% vs. 69.23%, *p* = 0.014). Our findings align with several previous studies. For instance, a prospective RCT reported that AR achieved wider resection margins than NAR (*p* = 0.023) and significantly prolonged the time to first local recurrence (53 vs. 10 months; *p* = 0.01) [32]. Similarly, Minagawa et al. in a Japanese study of 250 patients using PSM demonstrated superior 5‐year RFS with AR (62% vs. 35%; *p* = 0.005) [41]. However, not all studies have reached the same conclusion, and some report no significant difference in recurrence‐free survival between AR and NAR [42].

Contrary to the traditional concern that AR may increase perioperative risks in cirrhosis due to the larger resection volume, a key finding of our study was the absence of significant differences in perioperative complications between AR and NAR groups. This finding held true for both cirrhotic and non‐cirrhotic patients across multiple perioperative metrics, including intraoperative blood loss, operative time, ICU admission rate, overall complication rate, and rates of major complications (e.g., liver failure, bile leakage, postoperative hemorrhage). This aligns with a large meta‐analysis of 43 studies, which found no significant difference in perioperative complications between AR and NAR (RR: 0.95, 95% CI: 0.81–1.11; *p* = 0.49) [43]. Nevertheless, conflicting results exist in the literature. For example, a multicenter European study reported a higher overall complication risk in the NAR group compared to the AR group, particularly for severe (Clavien‐Dindo grade III: 14% vs. 10%; grade IV: 23% vs. 5%) complications [44]. This discrepancy compared to most studies may stem from several factors. Firstly, the cohort comprised predominantly European patients, where HBV infection (the primary HCC etiology in our study) is less prevalent, and HCV/alcohol‐related etiologies are more common. Moreover, the smaller sample size (*n* = 298) and the lack of propensity score matching to balance baseline characteristics between groups may limit the robustness of their conclusions.

Despite its contributions, our study has several limitations. First, the retrospective design inherently carries risks of selection bias and unmeasured confounding, despite our use of PSM to address known confounders. Future prospective randomized controlled trials (RCTs), particularly focusing on cirrhotic patients, are needed to provide higher‐level evidence regarding the comparative efficacy and safety of AR versus NAR. Second, our study cohort was exclusively Chinese, with hepatitis B virus (HBV) as the predominant etiology. We explicitly acknowledge that this HBV‐dominant profile may limit the generalizability of our findings, as hepatocellular carcinoma (HCC) arising from other causes such as hepatitis C virus (HCV) infection or alcohol abuse often exhibits distinct clinical profiles, disease progression patterns, and underlying molecular pathogenesis. These differences could influence postoperative outcomes and the effectiveness of surgical interventions. Therefore, external validation in cohorts where HCV or alcohol are the primary HCC risk factors is essential to confirm the broader applicability of our results. Third, our analysis did not stratify patients based on specific tumor characteristics (e.g., size, location, numbers). Future studies incorporating detailed tumor characteristics and potentially biomarkers could refine patient stratification to identify subgroups deriving maximal benefit from AR. This would facilitate personalized surgical decision‐making. Fourth, a further limitation lies in the interpretation of recurrence patterns. Due to the inherent limitations of a retrospective design, most patients lacked systematic follow‐up imaging, resulting in missing detailed information on specific recurrence sites. This constrains our ability to elucidate the precise biological mechanisms by which anatomical resection reduces recurrence risk. Although the available analysis suggests an association between anatomical resection and lower intrahepatic recurrence, this finding should be regarded as exploratory and requires validation through prospective studies with standardized imaging follow‐up.

## 5 Conclusion

In conclusion, the optimal surgical approach (AR vs. NAR) for HCC, especially in cirrhosis, remains debated. Our findings suggest that AR can be performed safely in carefully selected cirrhotic patients without increasing perioperative morbidity or mortality, given appropriate preoperative evaluation and meticulous perioperative care. Furthermore, AR demonstrated superior oncological outcomes, including improved overall and recurrence‐free survival, compared to NAR in both cirrhotic and non‐cirrhotic patients. This study contributes to the evolving evidence supporting AR as a viable and effective option for cirrhotic HCC patients, particularly for patients primarily caused by HBV infection.

## Author Contributions

**Ao Du:** conceptualization (lead), methodology (lead), software (lead), writing – original draft (equal), writing – review and editing (equal). **Hongwei Xu:** data curation (lead), formal analysis (lead), investigation (equal), writing – review and editing (equal). **Chuang Jiang:** supervision (lead), validation (equal), visualization (equal). **Kefei Yuan:** writing – project administration (lead), resources (lead), writing – review and editing (equal).

## Funding

This work was supported by grants from the Natural Science Foundation of China (82472700, 82472745, 82472365, 82373400, 82372660, 82272685, 82202260 and 82173248), the Project funded by China Postdoctoral Science Foundation (2022TQ0221), 1.3.5 project for disciplines of excellence, West China Hospital, Sichuan University (ZYGD22006), Scientific and Technological Innovation Ability Enhancement Project for Junior Faculties of Sichuan University (2024SCUQJTX043), the Sichuan University postdoctoral interdisciplinary Innovation Fund (10822041A2103).

## Ethics Statement

The study was approved by the ethics committee of West China Hospital of Sichuan University.

## Conflicts of Interest

The authors declare no conflicts of interest.

## Supporting information


**Figure S1.** Flowchart of all included and excluded patients.

**Table S1.** Baseline clinicopathological characteristics of the study cohort.

**Table S2.** Results of Rosenbaum bounds sensitivity analysis for cirrhotic patients after PSM.

**Table S3.** Results of Rosenbaum bounds sensitivity analysis for non‐cirrhotic patients after PSM.

## Data Availability

All data included in this study are available upon request by contact with the corresponding author.
