# Supplementary figures and images for: Survival Benefit and Safety of Anatomic Resection in Cirrhotic Hepatocellular Carcinoma: Propensity‐Matched Analysis of 1699 Patients
Source: Cancer Med. 2026 Jan 23;15(1):e71537. doi: 10.1002/cam4.71537 (PMC12828670; doi:10.1002/cam4.71537)

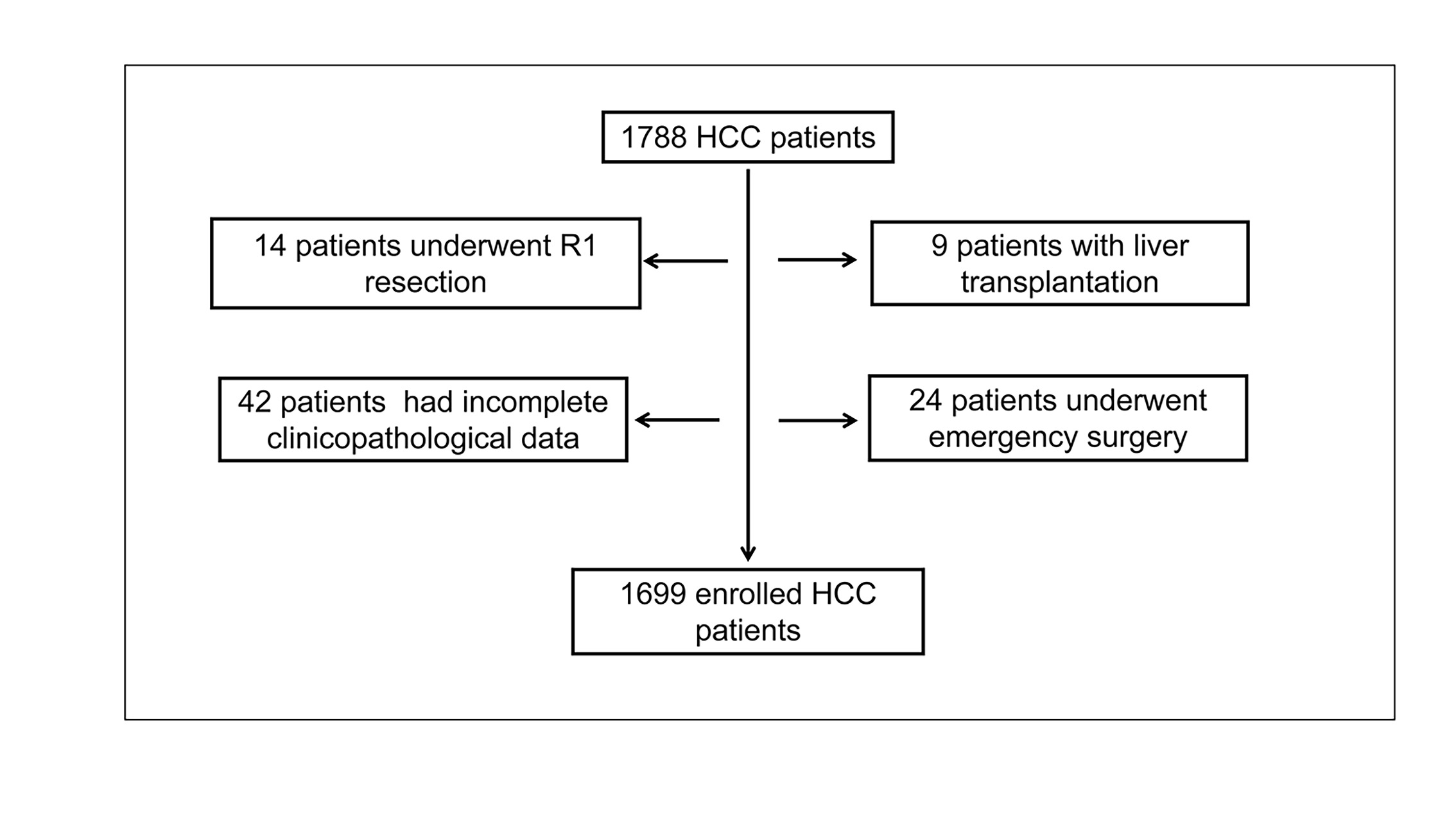

Supplement: Supplementary file 1 — Figure S1. Flowchart of all included and excluded patients. [file CAM4-15-e71537-s002.jpg]
