# Supplementary material for: Survival Benefit and Safety of Anatomic Resection in Cirrhotic Hepatocellular Carcinoma: Propensity‐Matched Analysis of 1699 Patients
Source: Cancer Med. 2026 Jan 23;15(1):e71537. doi: 10.1002/cam4.71537 (PMC12828670; doi:10.1002/cam4.71537)
Supplement: Supplementary file 2 — Table S1. Baseline clinicopathological characteristics of the study cohort. [file CAM4-15-e71537-s004.docx]

**Table S1. Baseline clinicopathological characteristics of the study cohort.**

| **Variables** | **Total (n=1699, %)** |
| --- | --- |
| Gender, n(%) |  |
| Male | 1442 (84.9) |
| Female | 257 (15.1) |
| Age (years), n(%) |  |
| ≤60 | 1211 (71.3) |
| ＞60 | 488 (28.7) |
| BMI(kg/m2) ,(median(IQR)) | 22.95 (20.81, 25.22) |
| Diabetes |  |
| No | 1563 (92.0) |
| Yes | 136 ( 8.0) |
| Hypertension, n(%) |  |
| No | 1436 (84.5) |
| Yes | 263 (15.5) |
| Cardiovascular disease, n(%) |  |
| No | 1671 (98.4) |
| Yes | 28 ( 1.6) |
| HBV infection, n(%) |  |
| No | 284 (16.7) |
| Yes | 1415 (83.3) |
| HCV infection, n(%) |  |
| No | 1674 (98.5) |
| Yes | 25 ( 1.5) |
| Alcohol, n(%) |  |
| No | 1003 (59.0) |
| Yes | 696 (41.0) |
| NAFLD, n(%) |  |
| No | 1580 (93.0) |
| Yes | 119 ( 7.0) |
| Cirrhosis, n(%) |  |
| No | 833 (49.0) |
| Yes | 866 (51.0) |
| AFP, n(%) |  |
| ≤400ng/ml | 1427 (84.0) |
| ＞400ng/ml | 272 (16.0) |
| Child-Pugh stage, n(%) |  |
| A | 1685 (99.2) |
| B | 14 (0.8) |
| Hepatectomy range, n(%) |  |
| Minor | 1259 (74.1) |
| Major | 440 (25.9) |
| Laparoscope, n(%) |  |
| No | 1368 (80.5) |
| Yes | 331 (19.5) |
| Intraoperative bleeding(ml), (median (IQR)) | 200.00 (100.00, 400.00) |
| Operation time(min), (median (IQR)) | 208.00 (165.00, 260.00) |
| ASA classification,, n(%) |  |
| 1 | 1128 (66.4) |
| 2 | 510 (30.0) |
| 3 | 61 (3.6) |
| Resection type, n(%) |  |
| AR | 618 (36.3) |
| NAR | 1081 (63.6) |
| Tumor size, n(%) |  |
| ≤5cm | 873 (51.4) |
| ＞5cm | 826 (48.6) |
| Tumor number, n(%) |  |
| Single | 1420 (83.6) |
| Multiple | 279 (16.4) |
| Macrovascular invasion, n(%) |  |
| No | 1556 (91.6) |
| Yes | 143(8.4) |
| MVI, n(%) |  |
| No | 1187 (69.9) |
| Yes | 512 (30.1) |
| Satellite, n(%) |  |
| No | 1545(90.9) |
| Yes | 154 (9.1) |
| Major complication (%) |  |
| No | 1609 (94.7) |
| Yes | 90 ( 5.3) |

AFP,α-fetoprotein; AR,anatomical resection; BMI,Body Mass Index; HBV,hepatitis B virus; HCV, hepatitis C virus; NAFLD, Non-alcoholic fatty liver disease; ASA,American Society of Anesthesiologists; MVI,microvascular invasion; NAR,nonanatomical resection.
