# Supplementary material for: Survival Benefit and Safety of Anatomic Resection in Cirrhotic Hepatocellular Carcinoma: Propensity‐Matched Analysis of 1699 Patients
Source: Cancer Med. 2026 Jan 23;15(1):e71537. doi: 10.1002/cam4.71537 (PMC12828670; doi:10.1002/cam4.71537)
Supplement: Supplementary file 3 — Table S2. Results of Rosenbaum bounds sensitivity analysis for cirrhotic patients after PSM. [file CAM4-15-e71537-s003.docx]

**Table S2. Results of Rosenbaum bounds sensitivity analysis for cirrhotic patients after PSM.**

| **OS** | | | | | **RFS** | | | |
| --- | --- | --- | --- | --- | --- | --- | --- | --- |
| Γ | Adjusted t-value | p value | Lower limit of significance level | Upper limit of significance level | Adjusted t-value | p value | Lower limit of significance level | Upper limit of significance level |
| 1 | 5.491 | <0.001 | <0.001 | <0.001 | 3.773 | <0.001 | <0.001 | <0.001 |
| 1.2 | 5.012 | <0.001 | <0.001 | <0.001 | 3.445 | <0.001 | <0.001 | 0.002 |
| 1.4 | 4.64 | <0.001 | <0.001 | <0.001 | 3.189 | 0.002 | <0.001 | 0.007 |
| 1.6 | 4.341 | <0.001 | <0.001 | <0.001 | 2.983 | 0.003 | <0.001 | 0.018 |
| 1.8 | 4.092 | <0.001 | <0.001 | 0.002 | 2.813 | 0.005 | <0.001 | 0.037 |
| 2 | 3.882 | <0.001 | <0.001 | 0.006 | 2.668 | 0.008 | <0.001 | 0.060 |
| 2.5 | 3.473 | <0.001 | <0.001 | 0.029 | 2.386 | 0.017 | <0.001 | 0.132 |
| 3 | 3.17 | 0.002 | <0.001 | 0.068 | 2.179 | 0.030 | <0.001 | 0.209 |
| 3.5 | 2.935 | 0.004 | <0.001 | 0.117 | 2.017 | 0.044 | <0.001 | 0.282 |
| 4 | 2.745 | 0.006 | <0.001 | 0.171 | 1.887 | 0.060 | <0.001 | 0.346 |
| 5 | 2.455 | 0.014 | <0.001 | 0.273 | 1.688 | 0.092 | <0.001 | 0.451 |
| 6 | 2.241 | 0.025 | <0.001 | 0.361 | 1.540 | 0.124 | <0.001 | 0.530 |
