# Supplementary material for: Survival Benefit and Safety of Anatomic Resection in Cirrhotic Hepatocellular Carcinoma: Propensity‐Matched Analysis of 1699 Patients
Source: Cancer Med. 2026 Jan 23;15(1):e71537. doi: 10.1002/cam4.71537 (PMC12828670; doi:10.1002/cam4.71537)
Supplement: Supplementary file 4 — Table S3. Results of Rosenbaum bounds sensitivity analysis for non‐cirrhotic patients after PSM. [file CAM4-15-e71537-s001.docx]

**Table S3. Results of Rosenbaum bounds sensitivity analysis for non-cirrhotic patients after PSM.**

| **OS** | | | | | **RFS** | | | |
| --- | --- | --- | --- | --- | --- | --- | --- | --- |
| Γ | Adjusted t-value | p value | Lower limit of significance level | Upper limit of significance level | Adjusted t-value | p value | Lower limit of significance level | Upper limit of significance level |
| 1 | 7.368 | <0.001 | <0.001 | <0.001 | 4.142 | <0.001 | <0.001 | <0.001 |
| 1.2 | 6.726 | <0.001 | <0.001 | <0.001 | 3.781 | <0.001 | <0.001 | <0.001 |
| 1.4 | 6.227 | <0.001 | <0.001 | <0.001 | 3.501 | <0.001 | <0.001 | 0.003 |
| 1.6 | 5.825 | <0.001 | <0.001 | <0.001 | 3.275 | 0.001 | <0.001 | 0.010 |
| 1.8 | 5.492 | <0.001 | <0.001 | <0.001 | 3.087 | 0.002 | <0.001 | 0.022 |
| 2 | 5.210 | <0.001 | <0.001 | <0.001 | 2.929 | 0.004 | <0.001 | 0.039 |
| 2.5 | 4.660 | <0.001 | <0.001 | 0.003 | 2.620 | 0.009 | <0.001 | 0.098 |
| 3 | 4.254 | <0.001 | <0.001 | 0.014 | 2.391 | 0.017 | <0.001 | 0.168 |
| 3.5 | 3.938 | <0.001 | <0.001 | 0.036 | 2.214 | 0.027 | <0.001 | 0.237 |
| 4 | 3.684 | <0.001 | <0.001 | 0.066 | 2.071 | 0.039 | <0.001 | 0.301 |
| 5 | 3.295 | 0.001 | <0.001 | 0.141 | 1.852 | 0.065 | <0.001 | 0.408 |
| 6 | 3.008 | 0.003 | <0.001 | 0.220 | 1.691 | 0.091 | <0.001 | 0.490 |
